# Case report: Using DNA short tandem repeats to confirm nongestational origin of pulmonary choriocarcinoma

**DOI:** 10.3389/fonc.2022.1001627

**Published:** 2022-10-17

**Authors:** Filipa Ferreira da Silva, Rita Barata, Inês Rolim, Catarina Carvalheiro, Nuno Gil, Marcos Pantarotto

**Affiliations:** ^1^ Gynaecology Unit, Medical Oncology Department, Champalimaud Foundation, Lisbon, Portugal; ^2^ Thoracic Tumors Group, Surgery Department, Champalimaud Foundation, Lisbon, Portugal; ^3^ Thoracic Tumors Group, Pathology Department, Champalimaud Foundation, Lisbon, Portugal; ^4^ Thoracic Tumors Group, Medical Oncology Department, Champalimaud Foundation, Lisbon, Portugal

**Keywords:** choriocarcinoma, lung neoplasms, primary tumor, non-gestational trophoblastic disease, human chorionic gonadotropin

## Abstract

Gestational trophoblastic neoplasias (GTN) are malignant neoplasms that occur in pregnant or recently pregnant women. Choriocarcinoma (CCA) is a highly aggressive and rare GTN, and cases outside the female genital tract are commonly seen as secondary manifestations of gynecologic disease. In this paper, we describe the case of a 40 years-old female patient with a primary pulmonary CCA who was surgically treated and for whom the confirmation of the primary origin of the tumor was possible using a DNA short tandem repeat genotyping. Distinction between gestational and non-gestational trophoblastic neoplasia is crucial as they require different therapeutic approach and have different prognoses.

## Introduction

Gestational trophoblastic neoplasias (GTN) are malignant neoplasms that occur in pregnant or recently pregnant women. Gestational choriocarcinoma (CCA) is a highly aggressive, malignant GTN consisting of cytotrophoblastic and syncytiotrophoblastic cells, distinct from other forms of malignancy for containing genetic material from the male partner. CCA can arise from any type of normal or abnormal pregnancy, and although its diagnosis generally occurs within months of pregnancy, it can be exceptionally present during gestation (1). The tumor typically secretes beta-human chorionic gonadotropin (β-hCG) (2).

CCA may have an exceedingly rare non-gestational origin in women. Extra-gonadal non-gestational CCA usually arises in midline structures such as the mediastinum, retroperitoneum, and central nervous system. Non-midline CCA is uncommon and mainly affects the lungs, gastrointestinal tract, or breast (3).

Few Primary Pulmonary CCA (PPC) cases are reported in the literature. They are generally metastatic and with poor prognosis. Here we report the case of a localized PPC, initially assumed as gestational CCA, where a DNA test confirmed the tumor’s non-gestational origin and ultimately guided our choices for the patient’s treatment.

## Case report

A 40-years-old female patient presented in our clinic in May 2021 for evaluation. She reported active smoking (20 packs-year) and had a history of secondary hypothyroidism. She had one uneventful vaginal delivery at age 31 and, since then, has been under continuous oral contraceptive.

In November 2020, she had a pregnancy diagnosis after a period of persistent nausea. One week later, she presented in the emergency room with spontaneous vaginal bleeding associated with pelvic pain. Physical examination was unremarkable. The analytic panel showed a β-hCG of 175U/mL (ref. value: <7.0U/mL), but pelvic ultrasonography (US) wasn’t compatible with pregnancy, suggestive of spontaneous abortion. In early December 2020, due to continuing complaints of pelvic pain and bleeding, she resorted to the ER again. Pelvic US suggested a right ectopic pregnancy and β-hCG was 200U/mL. A multidisciplinary decision was made for two courses of single-dose methotrexate (MTX) to be administered one month apart, with an initial reduction of β-hCG (nadir: 140U/mL) but subsequent elevation.

Due to failure of MTX therapy, surgical treatment was carried out with laparoscopy, bilateral salpingectomy, dilation and curettage procedures that showed no ectopic pregnancy, pelvic tumor, or pregnancy in the uterus. Her endometrium was in the proliferative phase, and biopsies from both salpinges showed no evidence of malignancy or scarring.

A whole-body ^18^F-FDG PET/CT scan showed a single hypermetabolic lesion (SUVmax 10.8) in the right upper lung lobe, measuring 24x32mm. A transthoracic needle biopsy of the lung lesion was consistent with CCA. No other lesions were identified after performing MRI of the brain, abdomen and pelvis, and thoracic CT scan.

According to the WHO/FIGO criteria (4) we assumed a low-risk GTN (4) with a single lung metastatic lesion with 3.2cm, who had not been treated with an appropriate MTX scheme. She started first-line chemotherapy (CTX) with MTX according to the 8-day Charing Cross regimen (5, 6), with persistent increase in β-hCG levels (maximum 1633U/mL). A CT scan performed after the second cycle of CTX revealed stability of the pulmonary lesion.

Considering the persistent elevation of the β-hCG despite the CTX, the absence of other foci of disease except for the lung and the fact that ectopic pregnancy has not been confirmed, a decision was made to treat the pulmonary lesion surgically.

**Figure 1:**
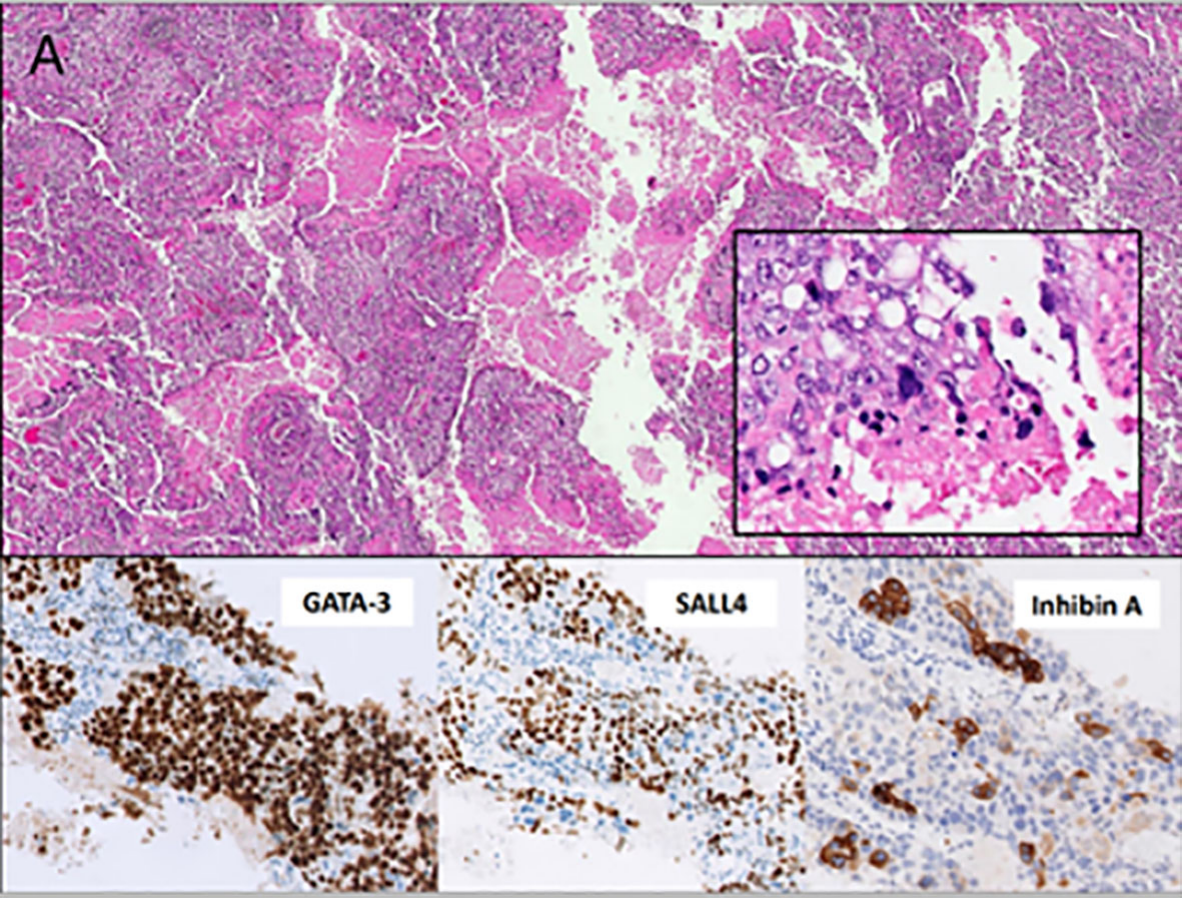
**(A)** The microscopic evaluation shows an extensively necrotic and hemorrhagic tumor with a distinctive biphasic appearance consisting of cytotrophoblasts, clear to light pink cytoplasm, distinct cell borders, irregular nuclei, and prominent nucleoli. Immunostaining of GATA-3 and SALL4, in close association with and capped by multinucleated syncytiotrophoblasts with abundant deeply eosinophilic cytoplasm, one or more nuclei, dark smudgy chromatin, and immunostaining of inhibin A and β-hCG (not shown). The neoplasm shows no immunostaining of TTF-1 (not shown).

In June 2021, or 8 months after the first symptoms, the patient had a thoracoscopic right upper lobectomy with systematic mediastinal lymphadenectomy. The postoperative course was uneventful, with the return of β-hCG titers to normal levels within three weeks. Histologic findings were consistent with CCA; immunochemistry showed positivity for β-hCG, Inhibin A, GATA3, SALL4, and negativity for TTF-1. Pathologic staging was pT2a pN0 R0 (stage IB, AJCC 8th ed) (**Figure 1**).

As a) there was no further evidence of more metastatic sites on the PET scan, b) the laparoscopy failed to reveal the initially supposed ectopic pregnancy in the absence of a molar pregnancy, and c) with the normalization of hCG hormone level after surgery according to the expected half-life of 24-36 hours, we considered the diagnosis of a non-metastatic PPC.

**Figure 2:**
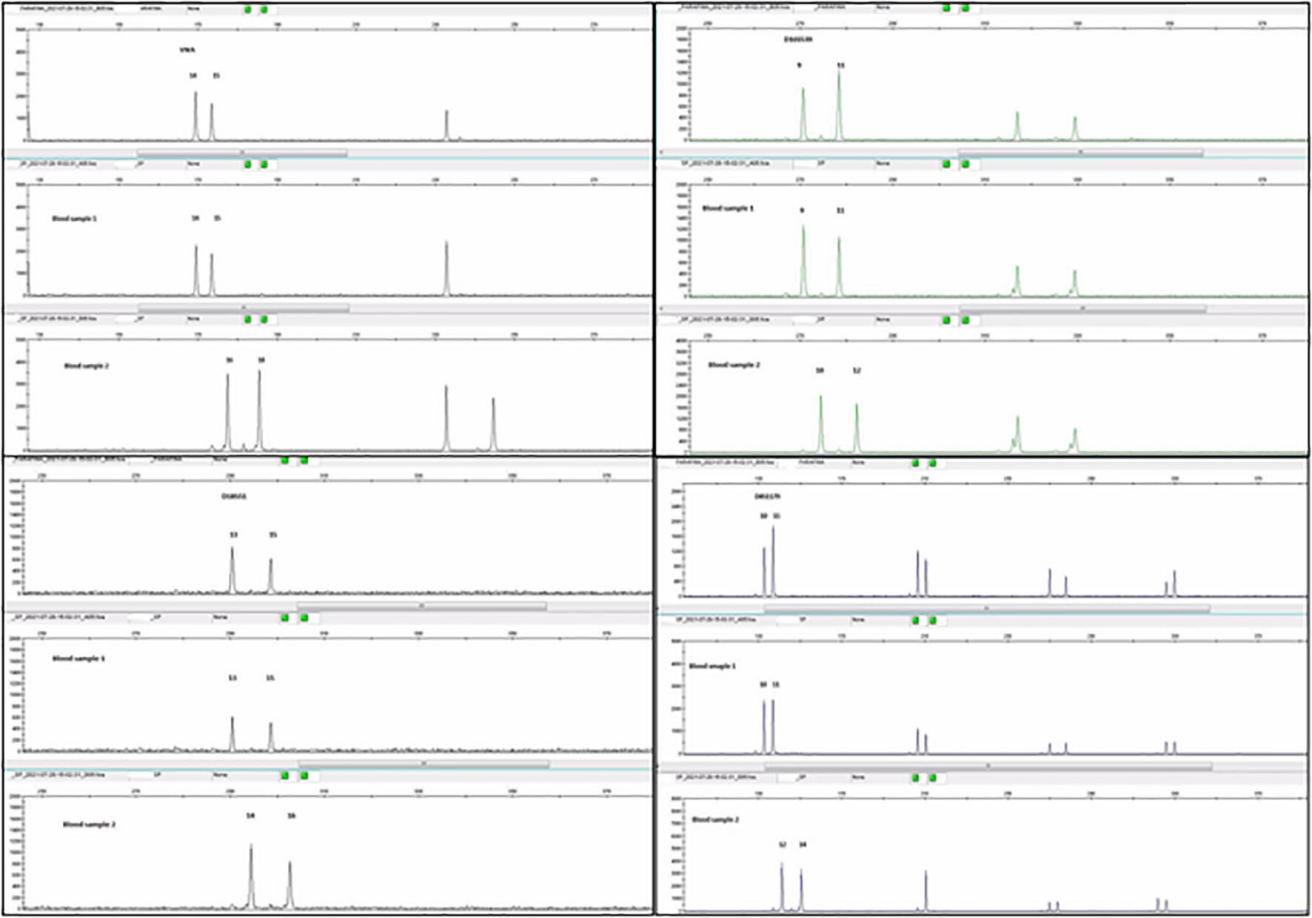
Comparative analysis of polymorphic markers (AmpFLSTR™ Identifiler™ Plus PCR Amplification Kit) in the tumor sample, patient (blood sample1) and partner (blood sample 2) blood samples. The profile of tumor´s and patient´s DNA are coincident, with no contribution from the partner`s DNA (in the case of informative markers) (image courtesy of Margarida Reis-Lima, MD, Elsa Garcia, MSc, Natália Salgueiro, MSc from Synlab laboratories, Porto, Portugal).

Being so rare, to confirm a non-metastatic PPC, we decided to analyze the genetic material of the tumor. A previously published case series used DNA’s short tandem repeats (STR) to investigate the non-gestational origin of CCA (7). For short tandem repeat genotyping a formalin-fixed paraffin-embedded block containing tumor was selected, and patient and partner peripheral blood samples. By comparing STR from the tumor and peripheral blood samples from the patient and her husband, we observed a tumor DNA profile consisting exclusively of the patient’s genetic pool, with no other donor contribution (**Figure 2**). Based on these findings, PPC was the final diagnosis.

**Figure 3:**
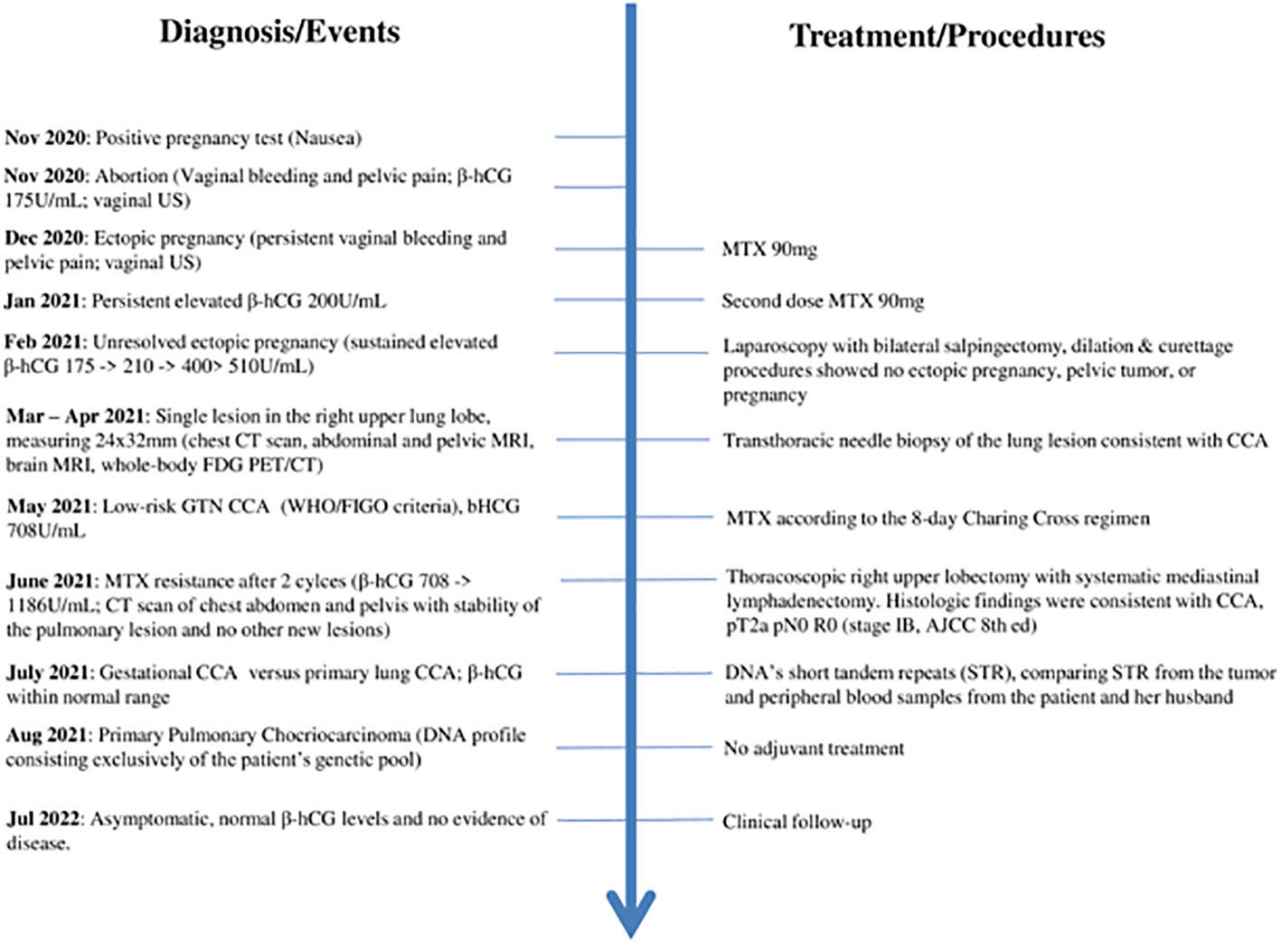
Timeline with relevant data from the episode of care. US – Ultrasound; MTX – methotrexate; GTN - Gestational trophoblastic neoplasia; CCA - choriocarcinoma.

No adjuvant treatment was offered, and after thirteen months (**Figure 3**), the patient is alive and asymptomatic, with normal β-hCG levels and no evidence of disease.

## Discussion

PPC is a very rare condition with a challenging diagnosis and management. The optimal therapy for PPC, especially in the early stages, is yet to be defined. The addition of chemotherapy after surgery has been suggested to improve survival, based on the aggressive nature of this rare cancer, and supported by a small series of patients (8). Nevertheless, in contrast to gestational CCA, PPCs are generally considered resistant to chemotherapy and carry a much poorer prognosis.

Differentiation between gestational choriocarcinoma and non-gestational choriocarcinoma can be very difficult since the clinical presentation and histology of these two diseases are identical. This differentiation is essential in order to establish the best therapeutic strategy.

In the case presented, with the initial suspicion of a recent abortion and subsequently of an ectopic pregnancy (refuted by laparoscopic examination), we considered the hypothesis of a metastatic lung lesion from gestational CCA as a more probable diagnosis. In addition, we know that this disease has very good prognosis and a high cure rate with chemotherapy. In order to define treatment, we applied the FIGO staging and the WHO risk score (4), resulting in a low-risk GTN (FIGO III: score <7). Assuming that the previous MTX scheme for ectopic pregnancy was not the standard regimen to treat this disease, we offered the 8-day Charing Cross MTX regimen (5, 6). The patient did not respond to CTX, with rising β-hCG levels, but fortunately maintaining radiological stable disease. In the face of resistance to CTX and the possibility of being a PPC case and not a metastatic CCA, we recommended the surgical treatment with radical intent. Histologic findings were consistent with CCA, and once again we were faced with doubt, whether it was really a PPC or metastatic gestational CCA.

The critical step for subsequent clinical decisions, such as adjuvant treatment, was determining the relation of the tumor with a gestational event or its primary origin. Molecular analysis of tumor tissue to identify unique paternal genetic contribution from a prior gestational event provides a definitive diagnostic distinction between tumors of gestational and non-gestational origin. Short tandem repeats (STR) genotyping is currently the most applicable method of identity testing, including diagnosis of gestational trophoblastic disease (9).

As reported above the tumor’s DNA fully matched to the patient’s, with no contribution from her partner’s DNA. This established the diagnosis of PPC. There are no clear prognostic features nor guidelines to assist in clinical decisions of PPC treatment. PPC tends to grow rapidly and has high propensity to metastasize to other organs such as bone, liver, brain, spleen and contralateral lung (10). Most reported cases refer to treatment of early stages with surgery followed by adjuvant chemotherapy with BEP (bleomycin, methotrexate and cisplatin) or EMA-CO (etoposide, methotrexate, actinomycin D, cyclophospamide and vincristine).

In contrast to previously published cases, the clinical course of this patient’s tumor was indolent, with stable disease for about 8 months until surgery was performed. We discussed with the patient the possibility of adjuvant CTX but ended up adopting a follow-up strategy instead.

Looking at the patient’s trajectory, the gynecological symptoms presented by the patient contributed to the delay in the diagnosis of PPC. When in doubt, we emphasize the possibility of performing STR genotyping to establish the diagnosis.

Given the current availability of STR DNA analysis and the possible benefits for patients with cases like the one presented here, we suggest that an STR DNA analysis from the tumor, the patient and partner should be used to support the diagnosis of PPC (1, 11).

## Data availability statement

The original contributions presented in the study are included in the article/Supplementary Material. Further inquiries can be directed to the corresponding author.

## Ethics statement

The studies involving human participants were reviewed and approved by Champalimaud Foundation's local ethics committee. The patients/participants provided their written informed consent to participate in this study. Written informed consent was obtained from the individual(s) for the publication of any potentially identifiable images or data included in this article.

## Author contributions

All authors listed have made a substantial, direct, and intellectual contribution to the work, and approved it for publication.

## Acknowledgments

The authors want to thank Margarida Reis-Lima, MD, Elsa Garcia, MD, and the Synlab laboratories in Lisbon, Portugal, for their help and collaboration in this case.

## Conflict of interest

The authors declare that the research was conducted in the absence of any commercial or financial relationships that could be construed as a potential conflict of interest.

## Publisher’s note

All claims expressed in this article are solely those of the authors and do not necessarily represent those of their affiliated organizations, or those of the publisher, the editors and the reviewers. Any product that may be evaluated in this article, or claim that may be made by its manufacturer, is not guaranteed or endorsed by the publisher.
